# Supplementary material for: Accuracy and interobserver-agreement of respiratory rate measurements by healthcare professionals, and its effect on the outcomes of clinical prediction/diagnostic rules
Source: PLoS One. 2019 Oct 3;14(10):e0223155. doi: 10.1371/journal.pone.0223155 (PMC6776326; doi:10.1371/journal.pone.0223155)
Supplement: S1 Questionnaire Dutch — (DOCX) [file pone.0223155.s007.docx]

**Kort onderzoek naar de ademhalingsfrequentie**

Graag willen we je vragen om mee te doen aan een kort elektronisch onderzoek naar de ademhalingsfrequentie.

Het doel van dit onderzoek is om te bepalen hoe goed de ademfrequentie bepaald kan worden. Wij zijn hierin geïnteresseerd, omdat deze parameter vaak gebruikt wordt om in te schatten hoe ziek een patiënt is. Verrassend genoeg is er nog maar weinig onderzoek gedaan naar de betrouwbaarheid van deze parameter!

Wij zouden het dan ook erg fijn vinden als je deze enquête zou willen invullen. Het invullen zal slechts 5 minuten duren.

Tijdens de enquête zal een aantal filmpjes getoond worden. Het is mogelijk om de filmpjes op ieder gewenst moment te stoppen. Om de filmpjes goed te zien kun je ze het best op een computer afspelen.

We vragen je de ademfrequentie in te schatten. Daarnaast stellen we je voorafgaand aan de filmpjes nog een paar vragen over je functie binnen de gezondheidszorg.

Voor het invullen van de meerkeuzevragen kan je klikken op het bolletje voor het antwoord van je keuze. Bij de open vragen moet je klikken op het tekstje 'Jouw antwoord', waarna je het antwoord in kan vullen.

Alvast heel erg bedankt voor je medewerking!

1. **Wat is je functie?**
   1. Student geneeskunde
   2. Co-assistent
   3. Semi-arts
   4. Verpleegkundige
   5. Ambulance verpleegkundige
   6. ICU verpleegkundige
   7. SEH verpleegkundige
   8. ANIOS
   9. AIOS
   10. Huisarts
   11. SEH-arts
   12. Andere medisch specialist, graag toelichting onder 'Anders'
   13. Anders
2. **Hoe lang heb jij deze functie in jaren (indien student, co-assistent of semi-arts: in welk studiejaar zit jij)?**
3. **Hoe meet jij de ademhalingsfrequentie normaal gesproken bij een patiënt?**
   1. 1 minuut meten
   2. 30 seconden meten
   3. 15 seconden meten
   4. 10 seconden meten
   5. Monitor meting
   6. Anders
4. **Zou je de ademhalingsfrequentie van de patiënt in het volgende filmpje (video 1) kunnen meten? (in aantal keer per minuut)**

**VIDEO 1**

1. **Hoe zou je de ademhalingsfrequentie van de patiënt in het vorige filmpje (video 1) beoordelen?**
   1. Verlaagd
   2. Normaal
   3. Verhoogd
2. **Zou je de ademhalingsfrequentie van de patiënt in het volgende filmpje (video 2) kunnen meten? (in aantal keer per minuut)**

**VIDEO 2**

1. **Hoe zou je de ademhalingsfrequentie van de patiënt in het vorige filmpje (video 2) beoordelen?**
   1. Verlaagd
   2. Normaal
   3. Verhoogd
2. **Zou je de ademhalingsfrequentie van de patiënt in het volgende filmpje (video 3) kunnen meten? (in aantal keer per minuut)**

**VIDEO 3**

1. **Hoe zou je de ademhalingsfrequentie van de patiënt in het vorige filmpje (video 3) beoordelen?**
   1. Verlaagd
   2. Normaal
   3. Verhoogd
2. **Zou je de ademhalingsfrequentie van de patiënt in het volgende filmpje (video 4) kunnen meten? (in aantal keer per minuut)**

**VIDEO 4**

1. **Hoe zou je de ademhalingsfrequentie van de patiënt in het vorige filmpje (video 4) beoordelen?**
   1. Verlaagd
   2. Normaal
   3. Verhoogd
2. **Zou je de ademhalingsfrequentie van de patiënt in het volgende filmpje (video 5) kunnen meten? (in aantal keer per minuut)**

**VIDEO 5**

1. **Hoe zou je de ademhalingsfrequentie van de patiënt in het vorige filmpje (video 5) beoordelen?**
   1. Verlaagd
   2. Normaal
   3. Verhoogd
