# Supplementary material for: Accuracy and interobserver-agreement of respiratory rate measurements by healthcare professionals, and its effect on the outcomes of clinical prediction/diagnostic rules
Source: PLoS One. 2019 Oct 3;14(10):e0223155. doi: 10.1371/journal.pone.0223155 (PMC6776326; doi:10.1371/journal.pone.0223155)
Supplement: S1 Questionnaire English — (DOCX) [file pone.0223155.s008.docx]

**Short questionnaire regarding respiratory rate**

We would like to ask you to participate in a short electronic study regarding the respiratory rate.

The purpose of this study is investigating how well the respiratory rate can be measured. This topic has caught our interest, as this parameter is often used to determine the degree of a patient’s illness. Surprisingly enough, research on the reliability of the respiratory rate is scarce!

Participation in this questionnaire will only take 5 minutes.

In the questionnaire, several movies will be shown. It is at any time possible to pause a movie. In order to view the movies in best quality, we advise you to use a desktop computer.

Questions are asked regarding the respiratory rate, for each individual video. Prior to the videos, several questions will be asked about your current profession.

Multiple choice questions can be answered by clicking the bullets in front of your answer. Open questions can be answered by typing in the textbox “your answer”.

We thank you in advance for participation in this study!

1. **What is your current profession?**
   1. Medical student
   2. Intern
   3. Final year intern
   4. Nurse
   5. First responder
   6. ICU nurse
   7. Emergency Medicine nurse
   8. Resident, not in training for specialty
   9. Resident, in training for specialty
   10. General practitioner
   11. Emergency Medicine consultant
   12. Other medical specialist
   13. Other
2. **How many years of experience do you have in your current profession?**
3. **What is your preferred method of measuring the respiratory rate in a patient?**
   1. Measuring for 1 minute
   2. Measuring for 30 seconds
   3. Measuring for 15 seconds
   4. Measuring for 10 seconds
   5. Monitor values
   6. Other
4. **Please measure the respiratory rate in video 1 below (breaths/minute)**

**VIDEO 1**

1. **How would you rate the respiratory rate in video 1?**
   1. Low
   2. Normal
   3. High
2. **Please measure the respiratory rate in video 2 below (breaths/minute)**

**VIDEO 2**

1. **How would you rate the respiratory rate in video 2?**
   1. Low
   2. Normal
   3. High
2. **Please measure the respiratory rate in video 3 below (breaths/minute)**

**VIDEO 3**

1. **How would you rate the respiratory rate in video 3?**
   1. Low
   2. Normal
   3. High
2. **Please measure the respiratory rate in video 4 below (breaths/minute)**

**VIDEO 4**

1. **How would you rate the respiratory rate in video 4?**
   1. Low
   2. Normal
   3. High
2. **Please measure the respiratory rate in video 5 below (breaths/minute)**

**VIDEO 5**

1. **How would you rate the respiratory rate in video 5?**
   1. Low
   2. Normal
   3. High
