# Supplementary material for: Accuracy and interobserver-agreement of respiratory rate measurements by healthcare professionals, and its effect on the outcomes of clinical prediction/diagnostic rules
Source: PLoS One. 2019 Oct 3;14(10):e0223155. doi: 10.1371/journal.pone.0223155 (PMC6776326; doi:10.1371/journal.pone.0223155)
Supplement: Attachment — Submitted filename: Response to reviewers.docx [file pone.0223155.s009.docx]

Prof. Raffaele Serra, M.D., Ph.D

Academic Editor

PLOS ONE

9 September, 2019

Dear Prof. Raffaele Serra,

Thank you for the opportunity to revise our manuscript: “Accuracy and interobserver-agreement of respiratory rate measurements by healthcare professionals, and its effect on the outcomes of clinical prediction/diagnostic rules”.

We appreciate the careful review and constructive feedback. We feel that the changes that we made have improved the manuscript substantially. Our response to the reviewer’s and editor’s comments are included in this letter.

Yours sincerely,

On behalf of all authors.

Gideon Latten

| **Nr.** | **Comment** | **Answer** |
| --- | --- | --- |
| 1. | Please ensure that your manuscript meets PLOS ONE's style requirements, including those for file naming. | We have corrected our manuscript and files, in order to meet PLOS ONE’s style requirements. |
| 2. | Please include all of the videos used in your study as Supplementary Information files. | The Supplementary Information files section now contains all 5 videos. |
| 3. | Please include in your Methods section more details on how participants were recruited, including what types of social networks were targeted (professional, general), what period of time was given for participants to respond, and how minimum sample size was determined. | We expanded the methods section in order to clarify the recruitment process. |
| 4. | Please include copies of the survey questions or questionnaires used in the study, in both the original language and English, as Supporting Information, or include a citation if they have been published previously. | Copies of the questionnaire questions were included as supplementary files. |
| 5. | Please provide additional details regarding participant consent. In the ethics statement in the Methods and online submission information, please ensure that you have specified whether consent was suitably informed (ie. the purpose of the study was explained to participants). | We provided additional details regarding participant consent, in order to clarify the recruitment process and participant consent. |
| 6. | Please include a caption for figure 1. | Caption was added. |
| 7. | Please include captions for your Supporting Information files at the end of your manuscript, and update any in-text citations to match accordingly. | Captions were added. |
